# Neurotrophic factors in the physiology of motor neurons and their role in the pathobiology and therapeutic approach to amyotrophic lateral sclerosis

**DOI:** 10.3389/fnmol.2023.1238453

**Published:** 2023-08-24

**Authors:** Wesley M. Stansberry, Brian A. Pierchala

**Affiliations:** ^1^The Department of Anatomy, Cell Biology and Physiology, Stark Neurosciences Research Institute, Indiana University School of Medicine, Indianapolis, IN, United States; ^2^Medical Neuroscience Graduate Program, Indiana University School of Medicine, Indianapolis, IN, United States

**Keywords:** neurotrophic factor, ALS, BDNF, GDNF, CNTF, motor neuron

## Abstract

The discovery of the neurotrophins and their potent survival and trophic effects led to great enthusiasm about their therapeutic potential to rescue dying neurons in neurodegenerative diseases. The further discovery that brain-derived neurotrophic factor (BDNF), ciliary neurotrophic factor (CNTF) and glial cell line-derived neurotrophic factor (GDNF) had potent survival-promoting activity on motor neurons led to the proposal for their use in motor neuron diseases such as amyotrophic lateral sclerosis (ALS). In this review we synthesize the literature pertaining to the role of NGF, BDNF, CNTF and GDNF on the development and physiology of spinal motor neurons, as well as the preclinical studies that evaluated their potential for the treatment of ALS. Results from the clinical trials of these molecules will also be described and, with the aid of decades of hindsight, we will discuss what can reasonably be concluded and how this information can inform future clinical development of neurotrophic factors for ALS.

## Introduction

**Figure 1:**
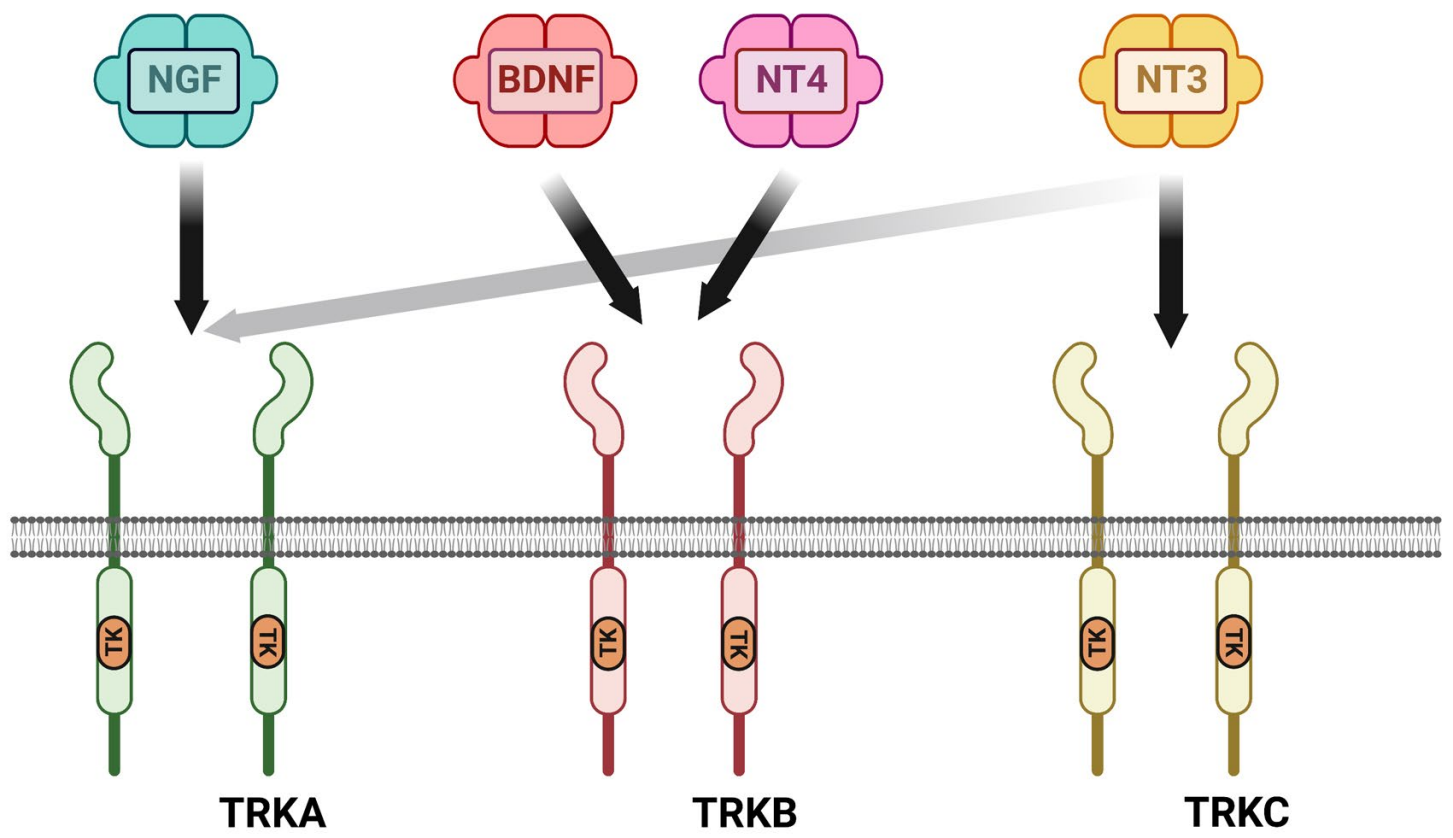
Neurotrophin receptor complexes. The neurotrophins consist of nerve growth factor (NGF), brain-derived neurotrophic factor (BDNF), neutotrophin-3 (NT3) and neurotrophin-4 (NT4). There are three receptor tyrosine kinases for the neurotrophins that convey survival and growth signals: TrkA is the receptor for NGF, TrkB is the receptor for BDNF and NT-4, and TrkC is the receptor for NT-3. NT-3 also binds to TrkA with lower affinity than TrkC.

**Figure 2:**
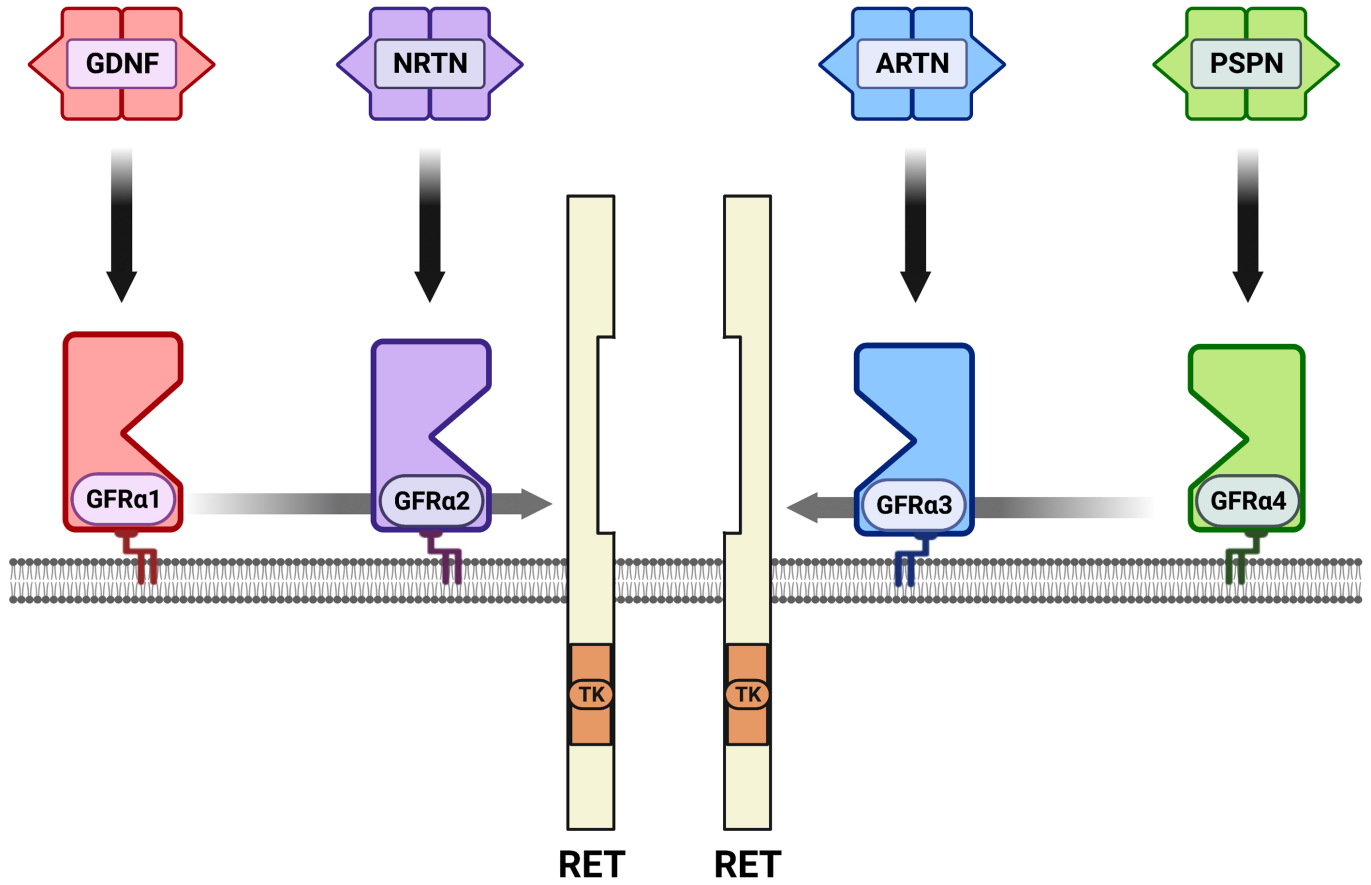
The glial cell line-derived neurotrophic factor (GDNF) family ligands (GFLs) and receptors. There are four secreted dimeric ligands named GDNF, neurturin (NRTN), artemin (ARTN) and persephin (PSPN) which bind with high affinity to glycerophosphatidylinositol-anchored surface receptors called GDNF family receptor alpha 1–4 (GFRα1-GFRα4). The high-affinity receptor for GDNF is GFRα1, GFRα2 for NRTN, GFRα3 for ARTN and GFRα4 for PSPN. There is some additional high affinity binding between ligands and GFRαs that is not shown; knockout studies support the preferred binding shown *in vivo*. All of the GFL-GFRα complexes then bind to the receptor tyrosine kinase RET, resulting in its dimerization, autophosphorylation and activation of the tyrosine kinase (TK) domain. In several cell types neuronal cell adhesion molecule (NCAM) serves as an alternative receptor to RET. Growth differentiation factor 15 (GDF15) is a distant family member that can activate RET as a complex with the GDNF family receptor alpha-like (GFRAL) surface receptor, not shown.

**Figure 3:**
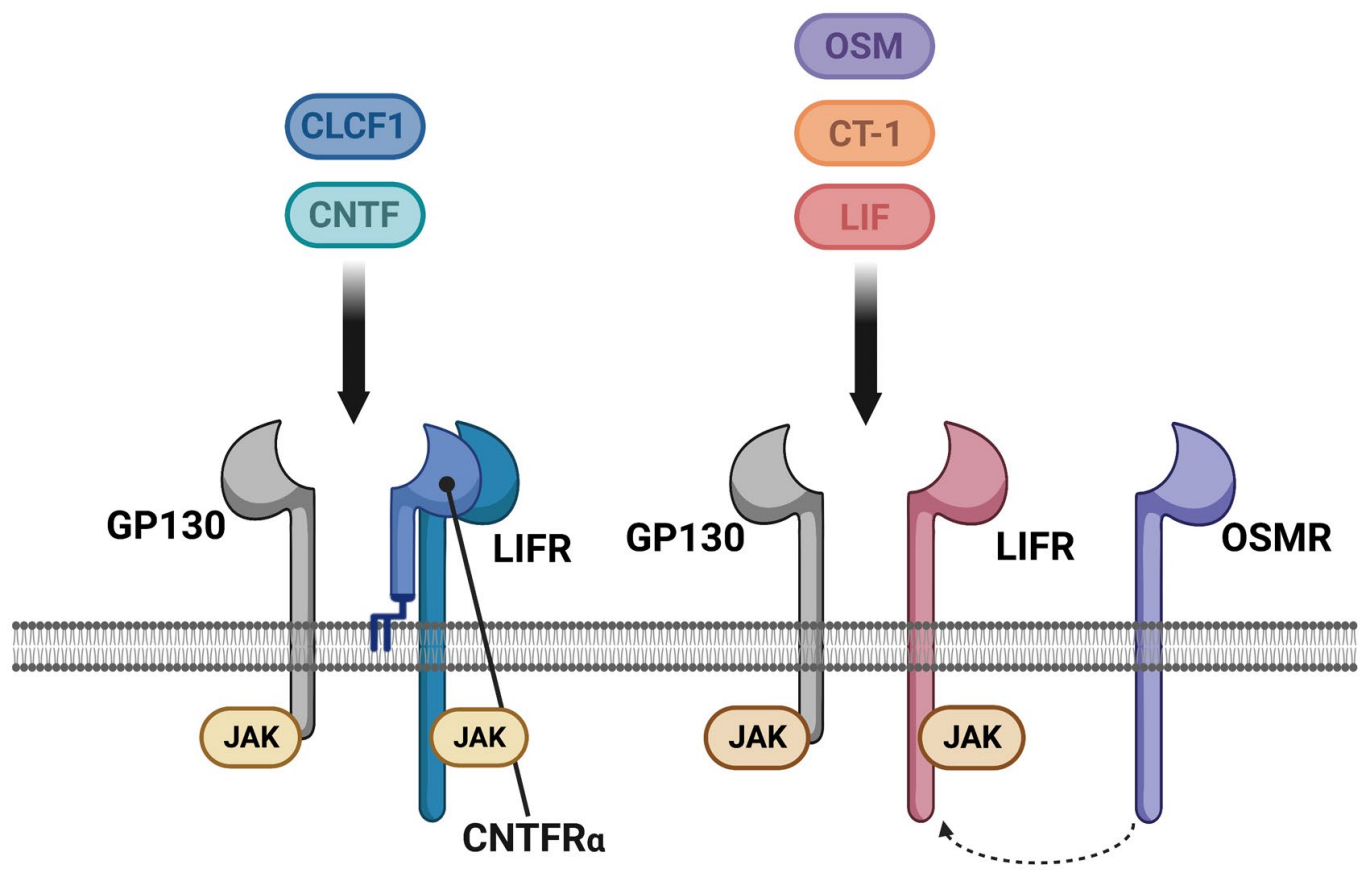
The cytokine family of neurotrophic factors and their receptors. Some cytokines act as neurotrophic factors, consisting of ciliary neurotrophic factor (CNTF), leukemia inhibitory factor (LIF), cardiotrophin-1 (CT-1), cardiotrophin-like cytokine factor-1 (CLCF1) and oncostatin-M (OCM). All five of these cytokines signal *via* two receptors, GP130 and LIF receptor (LIFR), which form a heterodimer when bound to the respective cytokine. CNTF and CLCF1 also associate with an additional co-receptor, the GPI-anchored CNTF receptor alpha (CNTFRα). In mice, but not humans or rats, OSM can also associate with the OSM receptor (OSMR) that is used instead of LIFR. Unlike the neurotrophins and GFLs that are dimers and therefore dimerize their receptor complexes, the cytokines function as monomers. GP130, LIFR and OSMR do not have an intrinsic tyrosine kinase domain, and are instead constitutively associated with intracellular JAK tyrosine kinases that are activated upon receptor engagement.

Neurotrophic factors are secreted growth factors that support the survival, cell fate specification, phenotypic maintenance and plasticity of neurons in both the CNS and PNS. The first family to be identified was the neurotrophins, consisting of nerve growth factor (NGF), brain-derived neurotrophic factor (BDNF), neurotrophin-3 (NT3) and neurotrophin-4 (NT4). The receptors that communicate these functions are the receptor tyrosine kinases known as the Trks. TrkA (*Ntrk1*) is the receptor for NGF, TrkB (*Ntrk2*) is the receptor for BDNF and NT4, and TrkC (*Ntrk3*) is the receptor for NT3 (Figure 1). Knockout studies in mice determined that the neurotrophins are critical for the target-dependent survival of peripheral neurons, including autonomic and sensory neurons, as well as motor neurons and numerous populations of CNS neurons. A second family of neurotrophic factors that is also critical for nervous system development and maintenance is the glial cell line-derived neurotrophic factor (GDNF) family ligands (GFLs) consisting of the founding member GDNF, neurturin (NRTN), artemin (ARTN) and persephin (PSPN). The four GFLs function predominantly via activation of the single receptor tyrosine kinase, Ret. GFLs do not bind to Ret directly, and instead form complexes with glycerophosphatidylinositol (GPI)-anchored receptors named GDNF family receptor alpha 1–4 (GFRα1-GFRα4), which show preferential affinity for specific GFLs (Figure 2; Donnelly and Pierchala, 2020). The GFLs are also critical for the development and maintenance of peripheral neurons, and have important functions for CNS neurons including spinal motor neurons, dopaminergic midbrain neurons and cerebellar neurons (Baloh et al., 2000; Airaksinen and Saarma, 2002). The third family of neurotrophic factors that this review will touch upon is the ciliary neurotrophic factor (CNTF) family of cytokines consisting of CNTF, leukemia inhibitory factor (LIF), oncostatin M (OSM), cardiotrophin-1 (CT1) and cardiotrophin-like cytokine factor (CLCF1), which are part of the larger IL-6 family of cytokines. These growth factors function *via* association with the two transmembrane receptor components gp130 and LIF receptor beta (LIFRβ), which engage the cytoplasmic JAK tyrosine kinases (Figure 3; Rose-John, 2018).

A receptor that is capable of binding to all four neurotrophins is p75, which is the subject of its own section later in this review. P75, when participating as part of a neurotrophin-TRK complex, increases the binding affinity of the complex, thereby enhancing survival activity. P75 can also bind to the neurotrophins in the absence of TRKs and, in this context, actually promotes apoptosis (Chao, 1992; Barker, 2004). Proneurotrophins, which retain the pro-domain required for protein folding while in transit through the secretory pathway, bind to p75 with a considerably higher affinity than the mature neurotrophins, and this complex also promotes degeneration and apoptosis (Lee et al., 2001; Hempstead, 2006). Surprisingly, p75 can also associate with GFL/GFRα/Ret signaling complexes and enhances survival signaling of Ret-expressing nociceptors during postnatal development (Chen et al., 2017; Donnelly and Pierchala, 2020).

Shortly after the discovery of the potent survival and growth effects of neurotrophic factors, they began to be evaluated as therapeutic molecules for the treatment of nervous system injuries and diseases. One of the first diseases to be considered was amyotrophic lateral sclerosis (ALS), also known as motor neuron disease (MND). ALS is a rapidly progressing, invariably fatal disease that causes the rather selective degeneration of upper and lower motor neurons, leading to fatal paralysis. While 10–15% of ALS cases are inherited, known as familial ALS (FALS), with many of the gene mutations identified, the remainder of ALS cases are sporadic (SALS), with no known genetic cause (Ferraiuolo et al., 2011; Zarei et al., 2015). Molecules from all three of these families of neurotrophic factors support the survival of motor neurons *in vitro* and have known functions in their maintenance *in vivo*. While there are other important growth factors that can support motor neurons, such as insulin-like growth factor 1 (IGF1) and vascular endothelial growth factor (VEGF), this review will predominantly focus on BDNF, GDNF and CNTF. This review will also discuss the transmembrane receptor p75 and its role in ALS.

### Brain-derived neurotrophic factor

The neurotrophins are composed of four secreted ligands, nerve growth factor (NGF), brain-derived neurotrophic factor (BDNF), neurotrophin-3 (NT3) and neurotrophin-4 (NT4). Their survival and growth promoting activities are communicated by the receptor tyrosine kinases known as the Trks; TrkA is the high affinity receptor for NGF, TrkB is the high affinity receptor for BDNF and NT4, and TrkC is the high affinity receptor for NT3 (Barbacid, 1994). BDNF was found to support the survival and growth of cultured spinal motor neurons isolated from rodents via activation of TrkB (Henderson et al., 1993; Wong et al., 1993), and this effect has subsequently been observed many times in various other experimental contexts, such as motor neurons differentiated from iPSCs (Lamas et al., 2014). Importantly, BDNF application in mouse models of nerve injury is neuroprotective to motor neurons (Koliatsos et al., 1993). Because of these potent survival effects *in vitro*, and their regenerative activity in injury models, even before the physiologic functions of the neurotrophins had been fully evaluated, there was enthusiasm for their potential use in the treatment of ALS (Seeburger and Springer, 1993).

The role of neurotrophin signaling pathways in spinal motor neuron development and maintenance is complex, as compared to peripheral neuron populations. Mice with germline deletion of *Trkb* demonstrated a significant loss of facial motor neurons, as much as 70%, a moderate loss of lumbar motor neurons, approximately 30%, but no loss of sacral motor neurons (Klein et al., 1993). While the varied survival effects of *Trkb* deletion was surprising, deletion of the TrkB ligands also yielded unanticipated results. Germline deletion of *Bdnf*, for example, demonstrated that it was not required for developmental survival of motor neurons (Jones et al., 1994). In fact, even *Bdnf*^*−*/−^; *Nt4*^−/−^ double knockout mice, the two TrkB ligands, have normal motor neuron numbers (Liu et al., 1995). It requires deletion of three neurotrophins in *Bdnf*^*−*/−^; *Nt4*^−/−^; *Nt3*^−/−^ mice to observe developmental motor neuron deficits, resulting in the loss of 20% of motor neurons and decreased somal size in the remaining motor neurons (Liu and Jaenisch, 2000). The observation that NT3 can have survival effects on motor neurons in the context of *Bdnf* and *Nt4* deletion is surprising, especially because mice lacking both *Trkb* and *Trkc* are not different from the individual knockout mice in regard to motor neuron development (Minichiello and Klein, 1996). Nevertheless, these studies on germline neurotrophin and *Trk* knockout mice revealed that there are likely redundant developmental survival mechanisms for motor neurons, which has also been observed for other populations of CNS neurons.

Perhaps more relevant for considering the therapeutic use of BDNF in ALS is whether BDNF/TrkB signaling is required for the maintenance or physiology of adult motor neurons. Conditional deletion models have been utilized for some these studies, and deletion of *Bdnf* selectively from muscle using HSA-Cre mice results in a phenotype shift of motor units from fast fatigueable (FF) to more slow (S) and fatigue resistant (FR) motor units (Delezie et al., 2019). There were not significant alterations in the integrity of NMJs, although they were generally smaller in *Bdnf*^f/f^; HSA-Cre mice (Delezie et al., 2019). This role of BDNF in FF physiology is consistent with the observations that BDNF, NT4, TrkB and phosphorylated TrkB (p-TrkB) are all higher in fast muscles, such as extensor digitorus longus (EDL) muscles and tibialus anterior (TA) muscles, as compared to soleus (slow) muscles (Just-Borras et al., 2022). The examination of whether TrkB is required for maintenance of motor neurons and their NMJs was evaluated in *Trkb*^+/−^ mice in which the TrkB dosage was reduced by 50% (Kulakowski et al., 2011). Interestingly, these mice showed premature aging of NMJs, with NMJs from 8-month-old *Trkb*^+/−^ mice looking similar to those of 24-month-old *Trkb*^+/+^ mice (Kulakowski et al., 2011). In *Trkb*^+/−^ mice, postsynaptic acetylcholine receptors had a broader, less dense organization with apparent denervation; there was, however, no apparent loss or atrophy of motor neuron cell bodies (Kulakowski et al., 2011). More recently, chemogenetic inhibition of TrkB *in vivo* has been utilized with *Trkb*^F616A^ knockin mice. The F616A point mutation renders TrkB sensitive to the kinase inhibitor 1NMPP1 at over 1,000-fold lower concentrations than wild type TrkB, allowing for application of 1NMPP1 to these mice to selectively inhibit TrkB^F616A^ (which is otherwise normal), and not affect other tyrosine kinases (Chen et al., 2005). Phrenic motor neurons, which are cervical motor neurons that innervate the diaphragm, were examined in this model, and 7–14 days of 1NMPP1 treatment caused some loss of NMJ integrity with reduced neurotransmission and a higher rate of muscular transmission failure (Mantilla et al., 1985; Fogarty et al., 2022). There was also a 16% loss of phrenic motor neurons in *Trkb*^F616A^ rats treated with 1NMPP1 for 2 weeks, suggesting that TrkB is necessary for some aspects of phrenic motor neuron physiology and survival in adulthood (Fogarty et al., 2023).

To begin to evaluate whether BDNF and TrkB may be involved in the progression of ALS, several studies have examined BDNF and TrkB expression in mouse *Sod1* mutant models and in ALS patients. In muscles of ALS mice, BDNF levels decreased as compared to wild type mice (Harandi et al., 2016), in contrast to another study in which BDNF increased in ALS mice (Just-Borras et al., 2020). In ALS patients, BDNF increased in muscle as compared to controls, but NGF, NT3 and NT4 all eventually increased in muscle at late-stage human disease as well (Kust et al., 2002). In spinal cord samples, BDNF expression did not appear to change in ALS patients compared to controls (Kawamoto et al., 1998). BDNF levels also did not change in the CSF of ALS patients, or ALS mice, as compared to controls (Grundstrom et al., 2000; Riolo et al., 2022). In serum isolated from blood, BDNF levels increased (Riolo et al., 2022), or were not significantly different (Tremolizzo et al., 2016; Cao et al., 2022) in ALS patients as compared to controls. In these two latter studies it was noted, however, that BDNF was the serum factor most changed in ALS patients, although not achieving significance (Cao et al., 2022), and that a reduced BDNF level did correlate with patients that had the worst revised ALS functional rating scale (ALSFRS-R) scores (Tremolizzo et al., 2016). TrkB levels in muscle from *Sod1*^G93A^ mice decreased in two independent studies (Harandi et al., 2016; Just-Borras et al., 2020). The level of TrkB increased in the postmortem spinal cords from ALS patients as compared to controls, but the level of its autophosphorylation was considerably lower than in controls, suggesting that while expression of TrkB was increasing, its level of activation was actually declining (Mutoh et al., 2000). These studies collectively do not present a consistent picture of BDNF and TrkB changes in ALS, and sometimes BDNF and TrkB levels change in opposite directions in the same studies, which has been interpreted as dysregulated or abnormal TrkB/BDNF signaling (Just-Borras et al., 2019; Rei et al., 2022). It should be noted that examining changes in BDNF and TrkB levels in isolation, whether in preclinical ALS models or in human patients, cannot definitively indicate whether these alterations are adaptive responses to disease or maladaptive responses that contribute to disease etiology.

An alternative means to evaluate BDNF/TrkB signaling in ALS is to examine retrograde transport of activated complexes during the progression of ALS, which has been done in ALS mouse models. Axonal transport deficits in general, both anterograde and retrograde, have been detected in preclinical ALS models, including the *Sod1*^G93A^ and *Tardbp43*^M337V^ mouse models of ALS (Perlson et al., 2009; Bilsland et al., 2010; Sleigh et al., 2020). Interestingly, transport deficiencies occur early in the disease process, prior to abnormalities in motor behavior or NMJ denervation (Bilsland et al., 2010). This could result in a loss of retrograde neurotrophic signals from BDNF/TrkB signaling complexes that were initiated in axon terminals, or in a loss of TrkB supplied to axon terminals from the cell bodies. A recent study determined that there is a selective reduction in the speed of retrograde transport of BDNF/TRKB complexes in fast motor neurons innervating the TA muscle of *Sod1^G93A^* mice, and no such reduction was seen in slow motor neurons innervating the Soleus muscle (Tosolini et al., 2022). Additionally, in fast motor neurons, injection of BDNF into the muscle increased the rate of retrograde transport, and this BDNF-dependent acceleration of transport was lost in fast motor neurons of *Sod1^G93A^* mice (Tosolini et al., 2022). This is interesting when one considers that slow motor neurons are more resistant to degeneration in ALS than fast motor neurons, which tend to have a larger motor unit size. Because BDNF/TrkB signaling appears to be more important in the phenotypic maintenance of FF motor neurons (Delezie et al., 2019), the greater changes in BDNF-dependent transport may be due to the higher level of TrkB signaling in these neurons. Reduced levels of phosphorylated TrkB have also been observed in spinal cords of ALS patients, raising the possibility that impaired transport of BDNF signaling complexes is occurring in human disease (Mutoh et al., 2000). While interesting, these studies on BDNF and TrkB have not been able to conclusively indicate whether BDNF/TrkB involvement in ALS is beneficial or harmful. A recent study identified two polymorphisms in the *BDNF* gene in humans that were correlated with ALS (Xu et al., 2017). While the sample size for this study was small and more studies are needed to confirm that these *BDNF* polymorphisms correlate with human ALS, determining whether they are loss or gain of function mutations would be an informative avenue of investigation (Xu et al., 2017).

While the most logical conclusion from these studies is that a loss of target-derived BDNF neurotrophic support contributes to the neurodegeneration in ALS, which would provide a rationale for the use of BDNF clinically, several studies raise further questions. In a study that evaluated whether TrkB serves a protective role in motor neurons in ALS mice, *Trkb^f/f^*; *Vacht*-Cre mice were produced in which *Trkb* was deleted selectively in postnatal motor neurons (Zhai et al., 2011). These mice were then crossed into *Sod1^G85R^* mice, a more slowly progressing mouse model of ALS. Counterintuitively, mice that had motor neuron deletion of *Trkb* were protected from neurodegeneration, and these *Trkb^f/f^*; *Vacht*-Cre; *Sod1^G85R^* mice had a considerably longer lifespan than *Trkb^f/f^*; *Vacht*-WT; *Sod1^G85R^* mice (Zhai et al., 2011). It is not fully understood how TrkB sensitizes motor neurons to ALS degeneration in *Sod1^G85R^* mice, but the answer could lie in the truncated form of TrkB that lacks the tyrosine kinase domain, TrkB.T1. TrkB.T1 typically acts in an inhibitory fashion to suppress TrkB signaling, and this splice variant is also deleted in *Trkb^f/f^*; *Vacht*-Cre mice. Recent studies have indicated that TrkB.T1 is upregulated in peripheral axons and NMJs in *Sod1^G93A^* mice (Just-Borras et al., 2020, 2022). Furthermore, germline deletion of *Trkb.T1* specifically, and not full length *Trkb*, is neuroprotective in *Sod1^G93A^* mice (Yanpallewar et al., 2012). Surprisingly, in a follow-up study, motor neuron-specific deletion of *Trkb.T1* was not neuroprotective in *Sod1^G93A^* mice, indicating that the protective effects of *Trkb* deletion in motor neurons cannot fully be explained by cell autonomous inhibitory *Trkb.T1* function in motor neurons (Yanpallewar et al., 2021; Tessarollo and Yanpallewar, 2022). In cultured spinal motor neurons, TrkB inhibition protected, and BDNF treatment sensitized, neurons to toxicity induced by kainite treatment, or by overexpression of SOD1 with ALS mutations (Mojsilovic-Petrovic et al., 2006). This suggests that a potentially deleterious effect of BDNF/TrkB signaling in motor neuron excitotoxicity and degeneration is most likely cell autonomous, in contrast to the effects of Trkb.T1 (Mojsilovic-Petrovic et al., 2006).

Given the potent survival effects of BDNF on motor neurons, BDNF moved into clinical trials for ALS. Two routes of administration were evaluated, systemic delivery *via* subcutaneous injection and introduction of BDNF into the CSF with intrathecal infusion using a continuous flow mini-pump. Initial phase I/II trials demonstrated that both methods of delivery were well-tolerated and, in the intrathecal delivery, reduced adverse side effects (American Neurological Association, 1995; Ochs et al., 2000). A phase III trial was completed for systemic BDNF, and neither BDNF treatment group, one at 25 g/kg and the second at 100 g/kg, showed a clinically significant benefit (BDNF Study Group, 1999). It should be noted that in this study, post-hoc tests showed that patients with demonstrated bowel effects from BDNF treatment, or patients that had respiratory impairment at study onset, saw a benefit (BDNF Study Group, 1999). For intrathecal delivery, a phase III clinical trial was conducted, but no beneficial effects were reported in two smaller studies from this trial (Kalra et al., 2003; Beck et al., 2005). A caveat to the application of BDNF protein in these clinical trials is the poor tissue permeability of BDNF, which could reduce the effective dose that reaches the intended target neurons. An alternative delivery approach that has been investigated is the use of cells that secrete BDNF, which upon intrathecal delivery become incorporated into the spinal cord. Human umbilical cord mesenchymal stem cells that are differentiated into motor neurons and that overexpress BDNF, upon intrathecal injection into *Sod1^G93A^* mice, improved motor control and increased lifespan as compared to injection of vehicle alone (Wang et al., 2021). Intrathecal injection of human motor neurons not expressing BDNF also showed motor and lifespan improvements, although not as significant as those expressing BDNF (Wang et al., 2021). On the other hand, neural progenitor cells overexpressing BDNF did not improve motor abilities or survival when implanted into spinal cords of *Sod1^G93A^* mice (Park et al., 2009), suggesting that the type of cell that is introduced, and how the transplantation is conducted, can significantly affect the success.

### Nerve growth factor

The potential involvement of NGF has been investigated in ALS in a limited number of studies, leaving its role in ALS unresolved. Several studies examined the expression of NGF in postmortem tissues of ALS patients, and in the *Sod1*^G93A^ mouse model. Expression of NGF in *Sod1*^G93A^ mice was reported as increased in the spinal cord by Western blotting (Kim et al., 2018), and more specifically in astrocytes by immunohistochemistry (Pehar et al., 2004). Astrocytes cultured from *Sod1*^G93A^ mice secrete NGF into their medium at greater levels than astrocytes from *Sod1*^WT^ mice, and conditioned medium from *Sod1*^G93A^ mice promotes degeneration of cultured motor neurons (Pehar et al., 2004; Ferraiuolo et al., 2011). In the analysis of postmortem spinal cord tissue from ALS patients, the published results are conflicting. NGF expression is reported to increase by qPCR and Western blotting (Nishio et al., 1998; Ferraiuolo et al., 2011), but decreased in an analysis of just the ventral horn by qPCR (Anand et al., 1995). NGF is reported to be upregulated in motor neurons of human postmortem ALS samples by immunohistochemistry (Nishio et al., 1998), although molecular markers of spinal motor neurons, such as ChAT, were not used to confirm that NGF-expressing cells were indeed motor neurons. Interestingly, post-translational modifications of NGF, such as nitration, that make NGF more likely to promote cell death, are increased in *Sod1*^G93A^ mice (Kim et al., 2018). NGF expression is also increased peripherally in muscle biopsies from ALS patients, compared to normal controls (Stuerenburg and Kunze, 1998; Kust et al., 2002), raising the possibility that NGF could act peripherally and/or centrally. Directly evaluating the involvement of NGF in the pathogenesis of ALS with blocking antibodies or genetic deletion in ALS models has not been reported.

Across these studies the hypothesis has been that NGF acts in a pro-degenerative manner *via* the p75 receptor, especially considering that motor neurons do not express TrkA and NGF is not a survival factor for them (Henderson et al., 1993; Wong et al., 1993; Bothwell, 1995). Interestingly, an analysis of neurotrophins and their receptors in postmortem human spinal cords suggested that motor neurons upregulate TrkA expression in ALS, proposing a potential switch in neurotrophin dependence from BDNF to NGF (Nishio et al., 1998). This was perhaps the justification for a recent clinical study evaluating intramuscular injection of NGF for the treatment of ALS (Li et al., 2022). The clinical trial was unusual in that the treatment course involved daily NGF injections for 28 days, followed by no treatment for 6 months, as opposed to continuous treatment used in other NGF clinical trials (Apfel et al., 2000). While the treatment course was reasonably well tolerated, there was no significant change in the progression of ALS between treatment groups (Li et al., 2022).

### Ciliary neurotrophic factor

Ciliary neurotrophic factor (CNTF) has long been known to play a neuroprotective role in several regions of the nervous system. First identified as a driver for ciliary ganglion neuron survival in chick embryos, several studies subsequently demonstrated CNTF expression in different types of neurons and glial cells, including reactive astrocytes and myelinating Schwann cells (Anand et al., 1995). Signaling through its tri-partite receptor complex comprised of CNTFR-α, LIFR-β and gp130, CNTF is involved in cytokine signaling cascades related to interleukin-6 and LIF (Ip et al., 1993). Interestingly, CNTFR-α can be released from cell surfaces, converting into a soluble form that can make the cells that do not express it responsive to CNTF in trans (Weis et al., 1998). In addition, CNTF promotes differentiation into a cholinergic neuron phenotype, and induces cholinergic genes such as choline acetyltransferase (ChAT) in primary sympathetic neurons (Saadat et al., 1989). Considering the heterogeneity of cell types expressing CNTF and the widespread expression of its receptors in the nervous system, CNTF remains a potential therapeutic target for neurological disorders.

CNTF was the first neurotrophic factor found to improve developing motor neuron survival after exogenous administration in both tissue culture and animal models, raising the possibility that it could be used as a potential treatment for motor neuron disorders (Anand et al., 1995). One study showed that although the deletion of CNTF does not appear to affect motor neuron development, the loss of CNTFR- α causes major motor deficits preventing jaw movement and suckling, causing mice to die perinatally (DeChiara et al., 1995). The first study that examined phenotypic deficits in response to CNTF deletion had more significant deficits, displaying motor neuron degeneration with progressive muscle weakness and atrophy in adult mice (Masu et al., 1993). A study quantifying CNTFR-α expression in denervated and dystrophic human muscle using *in situ* hybridization and northern blotting concluded that CNTFR-α expression in muscle is regulated by innervation, as expression was increased in atrophied fibers but not dystrophic ones (Weis et al., 1998).

Exogenous treatment with CNTF dramatically reduces degeneration in progressive motor neuropathy mice (Sendtner et al., 1992) and co-treatment of the wobbler mouse model of motor neuron disease with CNTF and BDNF completely arrests disease progression for 1 month (Mitsumoto et al., 1994). Using an organotypic spinal cord culture system, increased motor neuron sensitivity was observed in response to glutamate excitotoxicity when treated with CNTF. However, the authors of this study did highlight a number of potential confounding variables for using this culture system including the use of axotomized neurons that are missing the factors typically expressed and transported in motor axons (Corse et al., 1999). The ability of CNTF to drive motor neuron survival led to a series of studies investigating its involvement in ALS. A dual transgenic mouse model knocking out fibroblast growth factor 2 in *Sod1*^G93A^ mice showed that FGF-2 deficiency reduced motor neuron loss and extended survival and motor performance. Interestingly, CNTF expression was increased in gastrocnemius muscle in *Fgf2*^−/−^ mice, suggesting a potentially protective role for CNTF in ALS (Thau et al., 2012). This was supported by another study that demonstrated that CNTF deletion worsens motor phenotypes and reduces survival in the *Sod1*^G93A^ mouse model (Giess et al., 2002).

Although *in vitro* and *in vivo* models of CNTF treatment and involvement with motor neuron survival were clearly promising, studies involving humans have been less definitive. Immunostaining of postmortem brain and spinal cord tissues showed that CNTF protein is expressed in most upper and lower motor neurons in the CNS, and that this expression persists in ALS (Schorr et al., 1996). Conversely, *in situ* hybridization of the spinal cord and cerebral cortex for *Cntf* and *Cntfr*-α in postmortem tissues revealed no *Cntf* mRNA expression in either region, and that *Cntfr*-α was expressed only in lower motor neurons of ALS patients (Duberley et al., 1995). When examining CNTF levels in the spinal cord and cerebral cortex in ALS patients, there was a marked reduction in CNTF expression in the ventral horn of the spinal cord and no change in its expression in the cerebral cortex (Anand et al., 1995). Taken together, these results suggest that upper motor neurons do not express CNTFR themselves, and are not capable of responding to CNTF, suggesting that endogenous CNTF may not be involved in slowing the degeneration of upper motor neurons in ALS (Anand et al., 1995).

Serum CNTF levels measured in a study examining 96 familial and sporadic ALS patients revealed elevated CNTF in both ALS cohorts compared to inflammatory neurological controls and healthy controls. There was no correlation established, however, between CNTF levels and age of disease onset (Laaksovirta et al., 2008). One study looking at ALS patients with an inactivating *CNTF* polymorphism that results in truncated, non-functional CNTF protein concluded that the loss of CNTF expression did not increase susceptibility, or affect disease course, for either sporadic or familial ALS patients (Van Vught et al., 2007). A second study in 400 ALS patients and 236 controls that evaluated a prevalent *CNTF* null allele caused by a splice variant inducing a point mutation suggested that *CNTF* is not a significant disease modifier in ALS, as having the null allele appeared to make no difference in any aspect of disease progression. Furthermore, there appeared to be a delay in the age of onset of ALS in the homozygous *CNTF* null cohort (Al-Chalabi et al., 2003). A study analyzing postmortem tissues from 14 ALS patients and 13 controls that examined motor neurons via laser capture microdissection followed by microarray analysis observed that CNTF was upregulated in diseased motor neurons (Jiang et al., 2005). Partially supporting this result was a serum ELISA based study that concluded that CNTF serum levels were significantly higher for ALS patients compared to healthy controls. Interestingly, these elevated levels of CNTF failed to correlate with the clinical state of ALS patients’ disease onset or duration, leading to the study’s conclusion that while CNTF may be a reactive component of ALS, it cannot be used as a biomarker for ALS progression (Ilzecka, 2003).

Because of the early preclinical data supporting the neuroprotective activity of CNTF, clinical trials evaluated subcutaneous recombinant human CNTF (rhCNTF) as a potential treatment for ALS (Turner et al., 2001). In a phase 1 trial, single and repeated subcutaneous injections of rhCNTF with doses ranging from 2 to 100 μg/kg were administered to assess rhCNTF toxicity and tolerability (Miller et al., 1996). Adverse events ranged from mild reactions at the injection site to severe effects including re-activation of herpes simplex virus-related lesions and anorexia, correlating directly with increased dose amounts. The rhCNTF levels in CSF were undetectable for all dosage levels, raising the likelihood that upper motor neurons were not reached with subcutaneous treatment (Miller et al., 1996). Two larger clinical trials were done, and the first trial treated 730 patients for 9 months with three test groups: placebo, 15 μg/kg/day, or 30 μg/kg/day (ALS CNTF Treatment Study Group, 1996). Outcome measures included functional scale scores, isometric muscle strength and pulmonary function, and the rate of decline in muscle strength was taken as the primary endpoint. Both test groups compared to the placebo cohort showed no significant difference in outcomes or death rate (Turner et al., 2001). The second trial included 570 patients randomized between placebo, 0.5 μg/kg/day, 2 μg/kg/day and 5 μg/kg/day(Miller et al., 1996). Patients that completed the trial did not show significant improvements and, in fact, the 5 μg/kg/day cohort doubled in death rate during the trial (Turner et al., 2001). An alternative strategy for the delivery of CNTF was evaluated in another phase I clinical trial in which hamster kidney cells were genetically modified to express CNTF, encapsulated, and then surgically implanted into the lumbar intrathecal space. The capsule was placed in the CSF near the nerve roots of the cauda equina and tethered to the lumbodorsal fascia of ALS patients with the goal of releasing ~0.5 μg of hCNTF per day. Nanogram levels of CNTF were measured in the patients’ CSF for as long as measured, 17 weeks post-transplant, with no adverse side effects (Aebischer et al., 1996). Given that the use of CNTF for systematic treatment has been frustrated by the molecule’s short half-life, its inability to cross the blood brain barrier and the severity of peripheral side-effects, the results from this trial demonstrate a more promising method for continuously delivering CNTF to the CNS of patients in future trials (Aebischer et al., 1996).

### Glial cell line-derived neurotrophic factor

Ever since the initial discovery of GDNF and its survival-promoting effects on motor neurons, GDNF has been viewed as a potential therapeutic for ALS (Bespalov and Saarma, 2007). GDNF was originally identified as a neurotrophic factor capable of supporting the survival of dopaminergic midbrain neurons (Lin et al., 1993). It was subsequently discovered that GDNF supports the survival of motor neurons *in vitro* and is more effective than either BDNF or CNTF (Henderson et al., 1994; Oppenheim et al., 1995). Both *Gdnf*^−/−^ and *Ret*^−/−^ mice die shortly after birth due to kidney agenesis and a dramatic loss of enteric neurons (Schuchardt et al., 1994; Moore et al., 1996; Pichel et al., 1996; Sanchez et al., 1996), precluding the analysis of the nervous system at postnatal age. At birth, however, both *Gdnf*^−/−^ and *Ret*^−/−^ mice display a loss of lumbar spinal motor neurons of between 20 and 35% (Oppenheim et al., 1995; Moore et al., 1996; Sanchez et al., 1996). There are also axon pathfinding deficits in motor projections into the hindlimbs, as well as abnormalities in muscle innervation and terminal arborization (Haase et al., 2002; Livet et al., 2002; Kramer et al., 2006; Bonanomi et al., 2012). Interestingly, a careful analysis of germline and conditional *Ret* knockout models revealed that the loss of motor neurons was almost completely γ-motor neurons that innervate intrafusal muscle spindles (Gould et al., 2008). The innervation of muscle spindles by γ-motor neurons was also GDNF dependent (Whitehead et al., 2005; Gould et al., 2008). Therefore, GDNF-dependent Ret activation is critical for early developmental events in spinal motor neurons. It should be noted that *Gfrα1* mutant mice also display these developmental abnormalities, indicating that, in motor neurons, GDNF functions *via* GFRα1 (Gould et al., 2008). Interestingly, in mice in which *Ret* was deleted postnatally, thereby avoiding the early axon guidance and cell survival deficits, there is no alteration in the number of γ-motor neurons or α-motor neurons, indicating that Ret signaling is not required for the maintenance of spinal motor neuron survival in adulthood (Gould et al., 2008). It is possible, however, that GDNF/Ret pathways are necessary for structural maintenance of the NMJ, or for normal nerve-muscle communication. Indeed, it was recently shown that amyloid precursor protein (APP)-dependent GDNF expression is important for maintenance of NMJ innervation and force production postnatally, as *App*^−/−^ mice display deficiencies in both (Stanga et al., 2016).

Several studies have investigated whether GDNF, Ret, and/or GFRα1 expression is altered in mouse models of ALS and in postmortem tissues from ALS patients. Regarding GDNF, most studies that examined expression in muscle observed an increase in *Gdnf* mRNA and protein in muscle biopsies from ALS patients as compared to controls, or in the *Sod1*^G93A^ mouse model, although one study observed a decline (Yamamoto et al., 1996; Grundstrom et al., 1999; Yamamoto et al., 1999; Stanga et al., 2018). GDNF was either unchanged, or increased, in CSF samples from ALS patients as compared to controls (Grundstrom et al., 2000; Stanga et al., 2018), and conversely GDNF is reduced in blood serum (Stanga et al., 2018). Ret expression has been evaluated in spinal cord, and immunohistochemistry indicates that the majority of spinal motor neurons, greater than 85%, express Ret (Duberley et al., 1998). The available studies agree that Ret is either maintained, or only modestly reduced, in spinal cords of ALS patients compared to controls (Duberley et al., 1998; Mitsuma et al., 1999; Yamamoto et al., 2001; Zhang and Huang, 2006). The GFRα coreceptors have been evaluated both in spinal cord and muscle, and the available data suggests that GFRα1 and GFRα2 gradually decline with age in motor neurons of mice (Zhang and Huang, 2006). In muscle biopsies from ALS patients, however, GFRα1 levels are maintained (Yamamoto et al., 1999), and GFRα1 is also expressed at similar levels in spinal cord samples of ALS patients and controls (Mitsuma et al., 1999). Taken together, motor neurons appear to express the receptors necessary to respond to GDNF during the progression of ALS, which speaks to the feasibility of a potential therapeutic strategy. In addition, *Gdnf* levels generally appear to increase in muscle biopsies from ALS patients, and in muscles from *Sod1*^G93A^ mice, as compared to controls, but what conclusions can be drawn from these latter observations is unclear.

Evidence supporting a role for the GDNF/Ret signaling pathway in the pathobiology of ALS, either as an adaptive, neuroprotective pathway, or as part of the neurodegenerative process, is limited. A recent study evaluating the neuroprotective mechanism of the FDA-approved ALS drug Edaravone implicated GDNF and Ret in its neuroprotective effects. Using motor neurons differentiated from human iPSCs, it was determined that Edaravone was able to inhibit the neurotoxic effects of H_2_O_2_ and glutamate (Li et al., 2022). RNAseq identified GDNF and Ret as two of the most highly induced genes in response to Edaravone treatment of human motor neurons, and the combined treatment of H_2_O_2_-exposed neurons with Edaravone and GDNF completely prevented degeneration (Li et al., 2022), suggesting that GDNF/Ret pathway is part of the neuroprotective mechanism of Edaravone. In a study evaluating the role of the TNFα receptor TNFR1 in the progression of ALS, it was discovered that *Tnfr1*^−/−^ mice have a more rapidly progressing disease course when crossed into the *Sod1*^G93A^ line, as compared to *Tnfr1*^+/+^; *Sod1*^G93A^ mice (Brambilla et al., 2016). Stimulation of astrocytes with TNFα dramatically induces the expression of GDNF, and *Gdnf*, *Tnfα* and *Tnfr1* are all upregulated in the spinal cords of ALS patients, suggesting that GDNF induction by TNFR1 activation is an adaptive response during the progression of ALS (Brambilla et al., 2016). While most studies suggest that GDNF and Ret have a neuroprotective function on motor neurons, recent evidence suggests that inhibition of Ret tyrosine kinase activity may enhance retrograde transport pathways in motor neurons (Rhymes et al., 2022). Inhibition of Ret kinase activity with Compound C3 or LOXO-292, or Ret knockdown with siRNAs, accelerated endosome transport in cultured motor neurons, although this effect was not observed in sciatic nerve retrograde transport *in vivo* (Rhymes et al., 2022). GDNF treatment also accelerated retrograde transport dynamics in primary motor neurons *in vitro*, which is counterintuitive given that GDNF engagement of Ret would activate kinase activity, not inhibit it, arguing for the potential involvement of other signaling factors not examined in this study (Rhymes et al., 2022). Recently it was determined that Ret is capable of promoting apoptosis when it forms a complex with p75 upon its activation with pro-apoptotic ligands, whereas GFLs transition p75-Ret complexes to activate survival and growth pathways, suggesting that the transport-accelerating effects of Ret inhibition could be due to its functional signaling with p75 (Donnelly et al., 2018).

There are several studies that have evaluated whether GDNF can slow or prevent the degeneration of motor neurons in preclinical models of ALS, more than any other neurotrophic factor. In tissue culture models that evaluated motor neurons isolated from *Sod1*^G93A^ or *Sod1*^WT^ mice, or organotypic spinal cord slices exposed to excitotoxic injury, GDNF application to the medium was potently neuroprotective (Derby et al., 2000; Bilak et al., 2001). When moving to *in vivo* experiments using *Sod1*^G93A^ mice, *Gdnf* has been delivered using adenoviruses, lentiviruses and adeno-associated viruses (AAVs). GDNF has also been delivered using stem cells that were engineered to express and secrete GDNF. Regardless of how GDNF was ultimately delivered, studies have evaluated application into the spinal cord directly, into muscles in the periphery, or into the motor cortex to trigger the anterograde transport and release of GDNF into the spinal cord. Application of GDNF into the spinal cord to directly preserve motor neuron cell bodies has had variable results. Injection of lentivirus encoding *Gdnf* into the spinal cord of *Sod1*^G93A^ mice did not significantly alter the survival of motor neuron cell bodies, although injection into the facial motor nucleus did protect facial motor neurons (Guillot et al., 2004). Injection of human neural progenitor cells (hNPCs) expressing GDNF into *Sod1*^G93A^ mice resulted in effective engraftment of these cells, many differentiating into astrocytes, along with robust expression of GDNF (Klein et al., 2005; Suzuki et al., 2007). An additional benefit of using hNPCs is that naive astrocytes are neuroprotective in ALS, which is thought to be due to the ability of astrocytes to clear debris and provide a supportive microenvironment for motor neurons (Valori et al., 2023). Implantation of non-neural cell types, such as bone marrow or mesenchymal stem cells, into spinal cords of mouse models of disease is also neuroprotective, and it has been proposed that these effects are due to their secretion of neurotrophic factors such as GDNF (Pastor et al., 2012). In studies using hNPCs expressing GDNF, the chimeric region of the spinal cord that contained injected cells displayed markedly improved motor neuron survival as compared to untreated regions of the spinal cord, but NMJs still became denervated with disease progression, and the onset of disease was not delayed (Klein et al., 2005; Suzuki et al., 2007). Injection of human iPSCs encoding GDNF unilaterally into the lumbar spinal cord also preserved motor neurons, again without slowing disease progression (Laperle et al., 2023). Interestingly, delivery of human umbilical cord blood cells genetically engineered to express NCAM and GDNF together was more potently neuroprotective in the *Sod1*^G93A^ mouse model than the other methods just described (Islamov et al., 2015). In all of these spinal cord delivery studies, however, it is unclear to what extent significant improvements in motor performance and lifespan could be expected, given the application of cells to only one region of the spinal cord, sometimes unilaterally. Nevertheless, these efforts make clear that providing GDNF to motor neuron cell bodies in the spinal cord can support their survival, but may not result in the maintenance of NMJ innervation, thus not improving motor function or preventing muscle wasting in ALS.

**Figure 4:**
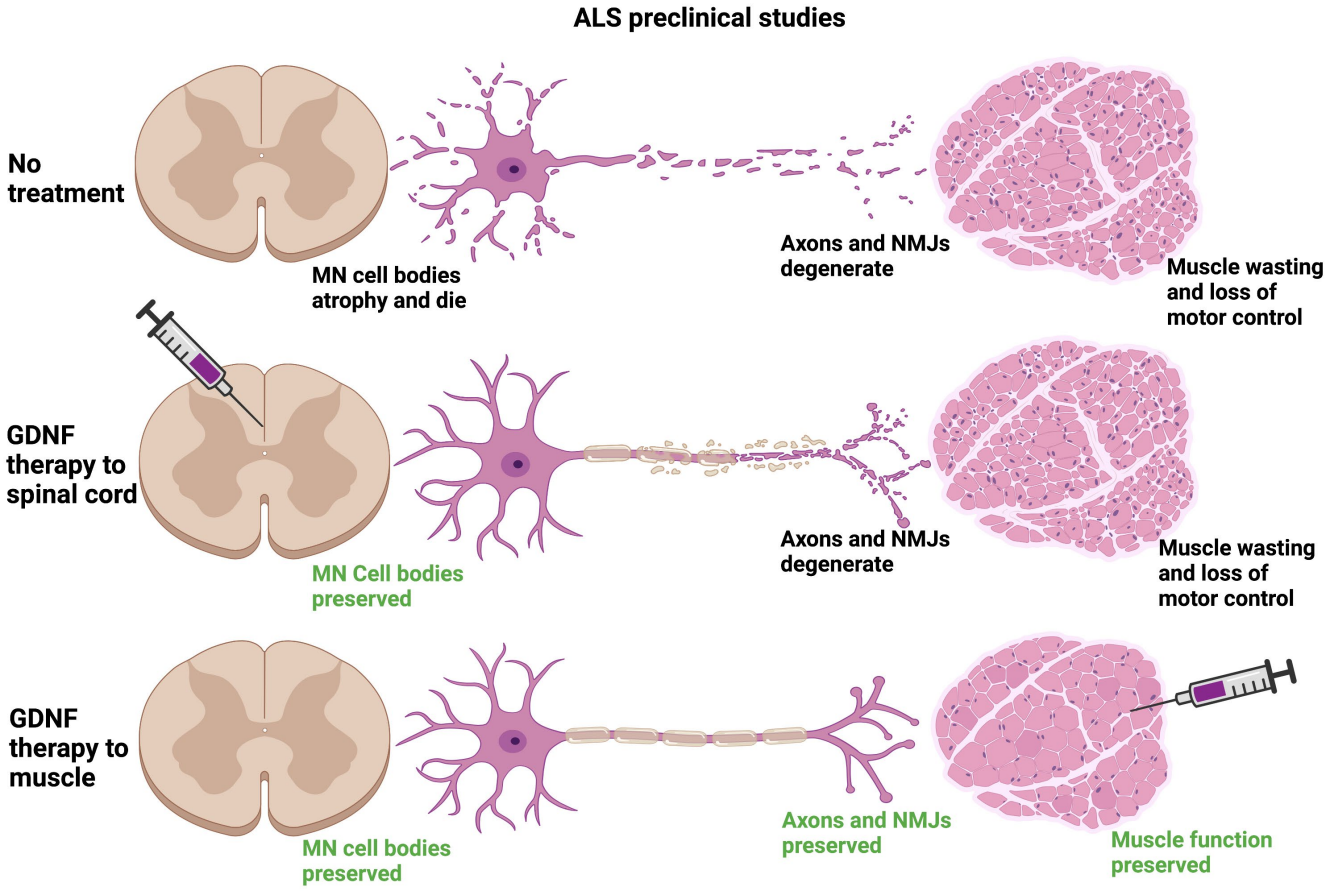
Preclinical data suggest that providing a source of GDNF in target muscle is neuroprotective to motor circuits degenerating in ALS. In mouse models of ALS, the *Sod1*^G93A^ model being the predominant model used for these studies, neuromuscular junctions (NMJs) are denervated along with degeneration of the motor axon. The motor neuron (MN) cell body also atrophies and MNs die. The loss of innervation at NMJs causes muscle wasting and a loss of motor control that is inevitably fatal (top row). Stem cells or lentiviruses expressing *Gdnf*, when provided intrathecally to the spinal cord, typically support motor neuron cell bodies in the vicinity of GDNF expression. In most studies these neuroprotective effects do not travel anterogradely to maintain NMJs and do not typically improve motor function or lifespan (middle row). When sources of *Gdnf* are provided to muscle, such as in viral vectors or myoblasts, the NMJs generally are maintained longer, leading to longer survival of MN cell bodies and improving lifespan (bottom row). Transgenic mice engineered to express *Gdnf* in muscle (Myo-*Gdnf*) show greater neuroprotection compared to GFAP-*Gdnf* mice that express *Gdnf* in astrocytes, consistent with these other studies.

In preclinical studies targeting GDNF expression in muscle, all three types of viral carriers very effectively drove GDNF expression in muscle for long periods of time, up to 2 months in some cases (Acsadi et al., 2002; Manabe et al., 2002; Wang et al., 2002; Lu et al., 2003). Viral-mediated *Gdnf* expression in muscle is more consistently neuroprotective, lengthening lifespan of *Sod1*^G93A^ mice for up to 17 days in two studies, as well as supporting increased survival of motor neurons innervating these muscles (Acsadi et al., 2002; Wang et al., 2002). It should be noted that the greatest increases in lifespan using intramuscular injection of *Gdnf*-encoding viruses were in studies that injected multiple muscles, both forelimb and hindlimb muscles (Acsadi et al., 2002; Wang et al., 2002). An alternative to viral-mediated expression of GDNF is to electroporate GDNF encoding plasmids into muscle, which did improve motor performance, although lifespan was unchanged (Yamamoto et al., 2001). Cells engineered to express GDNF have also been delivered to muscle, and both myoblasts and human mesenchymal stem cells (hMSCs) overexpressing GDNF supported motor neuron survival, NMJ innervation, motor function and increased lifespan (Mohajeri et al., 1999; Suzuki et al., 2008). Interestingly, transgenic mice that overexpress *Gdnf* under the myosin promotor (Myo-*Gdnf*) displayed a significant slowing of neurodegeneration in the *Sod1*^G93A^ model, but not GFAP-*Gdnf* mice that express *Gdnf* in astrocytes (Li et al., 2007). Taken together, GDNF expression in muscle appears to be more effective in slowing ALS neurodegeneration than delivery into the spinal cord (Figure 4), although how practical this would be for human clinical trials is unresolved.

A third delivery location is in the brain, and AAV5-*Gdnf* viruses have been injected into corticospinal tract or rubrospinal tract upper motor neurons (Foust et al., 2008). Interestingly, GDNF was observed in the lumber spinal cord in both delivery methods, although rubrospinal injections provided a greater anterograde transport of virally-encoded *Gdnf* (Foust et al., 2008). This delivery strategy has the advantage that it could promote the trophic support of both upper and lower motor neurons, both of which degenerate in ALS. Concordantly, transplantation of hNPCs encoding GDNF into the motor cortex of *Sod1*^G93A^ rats improved motor performance and lifespan as compared to injection of hNPCs not encoding GDNF (Thomsen et al., 2018).

From a practical standpoint, systemic delivery of viruses or stem cells encoding *Gdnf* would be the most clinically straightforward procedure. This was evaluated using AAV9-*Gdnf* that was injected into the tail vein of *Sod1*^G93A^ rats (Thomsen et al., 2017). This delivery method effectively drove GDNF expression in the spinal cord, cerebral cortex and in limb muscles. These mice showed an improvement in motor function tests such as grip strength, displayed increased spinal motor neuron survival and maintained NMJ innervation for longer (Thomsen et al., 2017). Lifespan was not significantly improved, however, and there were side effects from the widespread expression of GDNF, such as an impairment of working memory (Thomsen et al., 2017). In order to take advantage of systemic delivery, but still selectively express GDNF in muscle, AAV8 viruses were produced encoding *Gdnf* under the muscle-specific Desmin promoter (Modol-Caballero et al., 2021). This method resulted in the effective expression of GDNF in muscle and not in other tissues such as spinal cord, ultimately resulting in an increase in motor neuron survival, a longer maintenance of NMJ innervation, and significantly preserved electrophysiologic measures of neuromuscular function (Modol-Caballero et al., 2021). The ability to control transgene expression using specific promoters is a potentially powerful method for driving widespread expression of GDNF in skeletal muscle without requiring numerous intramuscular injections.

Similar to BDNF and CNTF, clinical trials using direct injection of GDNF protein were attempted for ALS. Subcutaneous and intrathecal administration of GDNF did not have therapeutic benefit in these trials, which was likely due to the short half-life of GDNF in blood, and the limited tissue diffusion of GDNF in spinal cord (Alisky and Davidson, 2000). A recent phase I/II clinical safety trial overcame these bioavailability problems with the use of hNPCs engineered to produce and secrete GDNF (Baloh et al., 2022). In this safety trial, the hNPCs^GDNF^ were injected into the lumbar spinal cord unilaterally, which proved to be well tolerated and resulted in GDNF expression in the injection regions (Baloh et al., 2022). Based on the preclinical studies indicating that cortical delivery of GDNF will be more effective than application into the spinal cord, a second clinical trial is underway evaluating delivery of hNPCs^GDNF^ into the motor cortex.

### P75NTR

The studies that suggest NGF acts in a degenerative manner as part of the etiology of ALS imply that p75 is the receptor communicating these prodegenerative signals. Several studies evaluated whether p75 expression is altered in ALS mouse models, and most report an increase in p75 expression in spinal cord. In both low-copy and high-copy *Sod1*^G93A^ mice, p75 expression is elevated in motor neurons compared to controls, although this increase was generally only seen in a small proportion of neurons (Lowry et al., 2001; Copray et al., 2003; Smith et al., 2015). P75 expression also increases in peripheral nerves of *Sod1*^G93A^ mice both in motor axons and in Schwann cells (Kerkhoff et al., 1991; Perlson et al., 2009). Consistent with these mouse studies, p75 is expressed in human ALS patient postmortem tissues in spinal motor neurons and in phagocytic Schwann cells in nerves (Seeburger et al., 1993; Lowry et al., 2001; Ferraiuolo et al., 2011; Trias et al., 2020). Upon ligand binding, p75 undergoes intramembranous cleavage resulting in the release of its extracellular domain (ECD)(Kanning et al., 2003). The ECD of p75 is elevated in the urine of ALS patients, raising the possibility of its use as a biomarker for ALS (Jia et al., 2017; Shepheard et al., 2017; Shi et al., 2021). Given its functions in apoptosis and axon pruning, it was hypothesized that inhibition or deletion of p75 would slow the progression of ALS. Female mice heterozygous for p75 (*p75*^+/−^) showed a modest protection when crossed with *Sod1*^G93A^ mice, but male mice were not protected (Küst et al., 2003). The interpretation of this study is limited, however, because *p75*^+/−^ mice do not have any deficits, suggesting there are no haploinsufficiency effects in heterozygous mice. Importantly, germline *p75*^−/−^ mice cannot be used in ALS studies because they develop a progressive peripheral neuropathy resulting in motor deficits (Lee et al., 1992). Preclinical studies have targeted p75 therapeutically using several methods, which were applied to the *Sod1*^G93A^ model, resulting in conflicting results. Antisense oligos targeting p75 resulted in modestly improved motor skills and extended lifespan when administered systemically (Turner et al., 2003). In contrast, two different inhibitors of p75 did not have neuroprotective effects in *Sod1*^G93A^ mice, although the efficacy of target inhibition, and dosing trials, were not conducted in these studies (Turner et al., 2004; Longo and Massa, 2013). Collectively these studies support the idea that p75 expression is increased in ALS, but no definitive conclusions can be drawn regarding the functional involvement of p75 in the pathogenesis of ALS, nor in whether inhibition of p75 has any effect in ALS models. The lack of preclinical therapeutic data from targeting p75 is perhaps not surprising, given that most methods used to target p75 so far inhibit both the pro-apoptotic and pro-survival effects of p75.

### Non-traditional neurotrophic factors

While most preclinical studies have evaluated BDNF, GDNF and CNTF, other growth factors have been explored as potential therapeutic molecules for motor neurons. Vascular endothelial growth factor (VEGF), insulin-like growth factor 1 (IGF-1), granulocyte-colony stimulating factor (G-CSF) and hepatocyte growth factor/scatter factor (HGF) have all been evaluated preclinically for ALS, and all of these molecules have undergone Phase I trials in humans. VEGF has been investigated in significant depth, partly because polymorphisms in the *VEGF* gene are associated with ALS in some studies, although not in others (Pronto-Laborinho et al., 2014; Da Costa et al., 2022). In addition, deletion of the hypoxia response element in the promoter of *Vegf* leads to motor neuron degeneration in mice, resulting in an ALS-like phenotype (Oosthuyse et al., 2001). The delivery of VEGF into *Sod1*^G93A^ mice is neuroprotective in some instances, such as intrathecal delivery of AAV9-*Vegf* viral vectors (Pronto-Laborinho et al., 2014; Wang et al., 2016). Just recently Phase I clinical trial results were reported for the delivery of VEGF into the lateral ventricle of ALS patients using minipumps, and this strategy was reasonably well tolerated (Van Damme et al., 2020). Another study evaluating the delivery of VEGF was conducted, trial NCT01384162, and results have not been published to our knowledge. In the case of HGF, its administration in preclinical models was shown to be neuroprotective, such as in a recent study that injected AAV6 encoding *Hgf* into hindlimb muscles (Lee et al., 2019; Desole et al., 2021). There have been human Phase I trials for HGF, using either intrathecal delivery of protein or intramuscular delivery of a plasmid encoding *Hgf*, and these studies confirmed safety (Sufit et al., 2017; Warita et al., 2019). For both VEGF and HGF, however, we await further clinical trials that will test their therapeutic efficacy in ALS.

There was significant interest in IGF-1 for the treatment of ALS, and numerous preclinical studies were conducted and Phase I trials were performed. This led to a Phase III trial that included 330 ALS patients that were treated with subcutaneous IGF-1 daily for 2 years. Unfortunately, this study concluded that there was no clinical benefit to IGF-1 treatment for ALS (Sorenson et al., 2008). Nevertheless, recent studies that evaluated systemic administration of scAAV9 encoding *Igf-1*, and intrathecal injection of IGF-1, observed a slowing of disease progression in mouse ALS models, raising the possibility that a different delivery method could be explored further (Hu et al., 2018; Wang et al., 2018). While G-CSF is best known for its effects on the immune system, it can also directly support the survival of several classes of CNS neurons, and preclinical experiments suggested it was neuroprotective in ALS models (Wallner et al., 2015). G-CSF (filgrastim) has been used clinically for years in leukemia patients, and its safety was confirmed in ALS trials (Iberl et al., 2019). A recent double-blind Phase II trial of filgrastim, the STEMALS-II trial, resulted in a 30% slowing in ALS symptoms by ALSFRS-R measures (Salamone et al., 2020). Whether this potential therapeutic benefit is due to the effect of G-CSF on the immune system, or more directly on the motor neurons or other CNS cell types, is still unknown.

## Conclusion

The initial enthusiasm for the use of neurotrophic factors in the treatment of ALS led to a series of clinical trials that showed little or no clinical benefit and, in the case of systemic administration of CNTF, some patients fared worse. These failures slowed further development of neurotrophic factors for the treatment of ALS, even though newer and more precise delivery methods are available. The preclinical development of GDNF has continued over the years, culminating in new clinical trials that were just recently initiated, which take advantage of transplantation of hNPCs engineered to express GDNF (Baloh et al., 2022). This same approach could be used for BDNF and/or CNTF, and a combination of hNPCs expressing multiple neurotrophic factors could potentially result in even more robust protective effects.

When these many studies on the preclinical development of BDNF, CNTF, GDNF and the non-traditional neurotrophic factors are considered in total, there are still many unresolved questions related to their potential effectiveness as ALS therapies. Nearly all of these ALS studies utilized the *Sod1*^G93A^ mouse model of ALS. While this is an exceptionally well-characterized model, the etiology likely differs from other ALS gene mutations. Analysis of the neuroprotective activity of BDNF, CNTF and GDNF in other available mouse models, such as *Tardbp*, *Fus* and *Ubqln2* mutation models that display motor neuron degeneration, would be prudent. In studies evaluating the effectiveness of neurotrophic factors in slowing the progression of the *Sod1*^G93A^ model, the most successful strategies only increase lifespan by 2–3 weeks. In addition, many of these studies began treatment at pre-symptomatic age in *Sod1*^G93A^ mice, which does not generally match when human ALS patients are diagnosed and begin treatment. Importantly, recently developed human iPSC motor neuron cultures, which allow for the evaluation of SALS motor neurons, should also be evaluated. Interestingly, BDNF, GDNF and CNTF all promote the survival of motor neurons derived from human iPSCs and are often included in the culture medium at the end stage of the differentiation process (Lamas et al., 2014).

On a more positive note, the therapeutic use of neurotrophic factors for ALS is more likely to be broadly effective for both FALS and SALS patients because it takes advantage of the intrinsic responsiveness of motor neurons to these factors, and does not rely on any one particular aspect of disease etiology. Indeed, when these factors, GDNF in particular, were successfully delivered to motor neurons in sufficient quantities, they were potently neuroprotective. Interestingly, the receptors themselves have been directly targeted for other conditions, such as the highly effective activating TrkB antibody (Guo et al., 2019), which represents an alternative strategy for activating neurotrophic signaling cascades. Likewise, small molecule activators of neurotrophic factor receptors, such as the Met receptor agonist K1K1 (Vallarola et al., 2020), or the non-peptide Ret modulator BT44 (Viisanen et al., 2020), have been developed for the treatment of neurodegenerative diseases. Small molecule receptor activators have the potential advantage that they may diffuse more readily through tissues and have a higher likelihood of crossing the blood brain barrier. In addition, more recently discovered neurotrophic factors, such as cerebral dopamine neurotrophic factor (CDNF), are being evaluated in preclinical ALS models, and CDNF recently was reported to slow progression of ALS in both *Sod1*^G93A^ and *Tardbp*^M377V^ mouse models (Lindahl et al., 2017; De Lorenzo et al., 2023). Another approach is to utilize multiple neurotrophic factors simultaneously. Along these lines, three clinical trials (two safety trials and one Phase III trial) were conducted using mesenchymal stem cells (MSCs) that were differentiated to secrete a mixture of neurotrophic factors (MSC-NTF cells). MSC-NTFs (NurOwn) were derived from the patient’s bone marrow, and were thus autologous transplantations. While intrathecal injection of NurOwn was found to be safe (Petrou et al., 2016; Berry et al., 2019), the phase III trial did not meet the clinical therapeutic endpoints, although a post-hoc analysis suggested that patients with less advanced ALS retained more motor function than the placebo group (Cudkowicz et al., 2022). In conclusion, given the progress that has been made in the delivery of secreted proteins into the CNS, with GDNF for ALS and Parkinson’s disease as an example, it may be an ideal time to more actively revisit neurotrophic factors for the treatment of ALS.

## Author contributions

WS and BP wrote and revised the manuscript. All authors contributed to the article and approved the submitted version.

## Funding

This work is supported by grant R01 NS089585 from the National Institutes of Health. WS is supported by NIH T32 AG071444 and BP is supported by NIH R01 NS089585. Schematic diagrams were made with BioRender software.

## Conflict of interest

The authors declare that the research was conducted in the absence of any commercial or financial relationships that could be construed as a potential conflict of interest.

## Publisher’s note

All claims expressed in this article are solely those of the authors and do not necessarily represent those of their affiliated organizations, or those of the publisher, the editors and the reviewers. Any product that may be evaluated in this article, or claim that may be made by its manufacturer, is not guaranteed or endorsed by the publisher.
